# Lepidosaurian diversity in the Mesozoic–Palaeogene: the potential roles of sampling biases and environmental drivers

**DOI:** 10.1098/rsos.171830

**Published:** 2018-03-21

**Authors:** Terri J. Cleary, Roger B. J. Benson, Susan E. Evans, Paul M. Barrett

**Affiliations:** 1 Department of Earth Sciences, Natural History Museum, Cromwell Road, London SW7 5BD, UK; 2 Department of Cell and Developmental Biology, University College London, Gower Street, London WC1E 6BT, UK; 3 Department of Earth Sciences, University of Oxford, South Parks Road, Oxford OX1 3AN, UK

**Keywords:** Lepidosauria, species-richness, Mesozoic, Palaeogene, subsampling, Grande Coupure

## Abstract

Lepidosauria is a speciose clade with a long evolutionary history, but there have been few attempts to explore its taxon richness through time. Here we estimate patterns of terrestrial lepidosaur genus diversity for the Triassic–Palaeogene (252–23 Ma), and compare observed and sampling-corrected richness curves generated using Shareholder Quorum Subsampling and classical rarefaction. Generalized least-squares regression (GLS) is used to investigate the relationships between richness, sampling and environmental proxies. We found low levels of richness from the Triassic until the Late Cretaceous (except in the Kimmeridgian–Tithonian of Europe). High richness is recovered for the Late Cretaceous of North America, which declined across the K–Pg boundary but remained relatively high throughout the Palaeogene. Richness decreased following the Eocene–Oligocene Grande Coupure in North America and Europe, but remained high in North America and very high in Europe compared to the Late Cretaceous; elsewhere data are lacking. GLS analyses indicate that sampling biases (particularly, the number of fossil collections per interval) are the best explanation for long-term face-value genus richness trends. The lepidosaur fossil record presents many problems when attempting to reconstruct past diversity, with geographical sampling biases being of particular concern, especially in the Southern Hemisphere.

## 1. Introduction

Lepidosauria consists of Rhynchocephalia, Squamata and a small number of stem taxa [1]. Today, Rhynchocephalia is reduced to a single genus, *Sphenodon*, the tuatara [2], whereas Squamata contains over 10 000 extant species of lizards, snakes and amphisbaenians [3]. Squamate body sizes range from those of tiny extant geckos such as *Sphaerodactylus* (less than 20 mm [4]) to the extinct mosasaurs (up to approx. 17 m in body length, although this is probably overestimated [5]). Members of the clade exhibit specializations for marine, fossorial and arboreal life, and exhibit wide variation in diet, behaviour and habitat. Historically, squamates acquired several features that have been regarded as key adaptations thought to contribute to their success, including limblessness [6,7], cranial kinesis [8] and venom-delivery apparatus [9]. They display varying methods of reproduction [10], including viviparity, oviparity, ovoviviparity and even parthenogenesis in some taxa [11]. As such, understanding patterns of taxic richness through time is relevant to understanding the origins of their exceptional extant richness.

There is continued debate over the timing of lepidosaur origins, but the confirmed presence of closely related groups (archosauromorphs), as well as evidence from molecular phylogenies suggests that it must have occurred by the Late Permian. Nevertheless, the fossil record currently indicates that the major radiation of the clade occurred much later (e.g. [7,12–16]). The earliest known rhynchocephalian fossils are from the Northern Hemisphere [1,17–19], but the absence of any record prior to the Middle Triassic obscures their geographical origins. A large temporal gap separates the earliest rhynchocephalian fossils (Ladinian [1]) from those of the earliest known squamates, limiting understanding of their early diversification. The earliest squamate fossils are from the Early or Middle Jurassic of India ([20]; though there is some uncertainty in the dating of these remains, [21]), and the Bathonian (Middle Jurassic) of the UK [22] and Central Asia [23]. Unequivocal snake fossils first appear in the Early Cretaceous [24–26] and might have had a Gondwanan origin given their wider distribution and higher species-richness in this region during their early evolution [27,28], although there is disagreement on the identification of older specimens from the Middle Jurassic as stem-group snakes [29]. Amphisbaenians, which are entirely fossorial, first appear in the early Paleocene of Belgium [30] and the USA [31], and the late Paleocene of Africa [32], though there are disputed Late Cretaceous findings [33]. Their fossil record is extremely sparse, and little is known about how they diversified, with modern species-richness concentrated in Africa and South America [34,35].

Although rhynchocephalians were abundant during the early history of lepidosaurs, their abundance and geographical distribution began to contract after the Early Jurassic. They made their final appearance in Asia during the Sinemurian despite the presence of younger microvertebrate localities that should be suitable for their preservation [28,36–38]. They subsequently continued to decline in the Northern Hemisphere, culminating in their last appearances in North America and Europe during the Early Cretaceous [36,39,40]. Although they are present in some Gondwanan localities in the Late Cretaceous, the Cenozoic fossil record of rhynchocephalians is sparse and, except for one occurrence in the Paleocene of Argentina [41], is confined to the area in which they are found currently, New Zealand [42]. Squamates, by contrast, expanded their ranges from their first appearances in the Jurassic of Europe and Asia to become near-cosmopolitan in the modern day, except in the coldest regions of the world, and are key organisms within the terrestrial ecosystems they inhabit [43].

Numerous studies have examined changes in species-richness in terrestrial lepidosaur communities around key events in the Earth history such as mass extinctions, but most of these have concentrated on the underlying causes of taxic richness change, rather than analysing the pattern of change itself (e.g. [41,44,45]). Some have reviewed large portions of the lepidosaur fossil record through time, for either small regions (e.g. [46–48]), whole continents (e.g. [49–51]) or globally [27,36], but these have been based primarily on qualitative observations and direct readings of the fossil record. However, many studies have now demonstrated that the fossil record offers an inherently incomplete view of the history of life, as it is unlikely that all taxa that ever lived have been fossilized, and only a proportion of those individuals that do become fossils will ever be discovered [52–54]. In addition, various biases can shift the observed richness away from the original ‘true’ richness, including rock outcrop availability, differences in preservation probability and anthropogenic sampling variability (see [54] for a summary). Few of the above-mentioned studies on the lepidosaur fossil record mention these inadequacies, and even fewer employ methods to account for these biases, with the exception of two recent studies on marine tetrapod diversity that include mosasaurs [55,56]. Beyond lepidosaur-focused research, there are now many studies that have examined the richness of the tetrapod [37,57,58] and the marine invertebrate [59–61] fossil records with regard to sampling biases in the rock record. While results differ, the majority of these larger-scale studies recover some significant correlations between various proxies representing sampling and/or geological biases and palaeobiodiversity, particularly for those representing rock availability or worker effort.

Many methods have been developed to untangle geological and sampling biases from the underlying ‘true’ taxon richness. These include comparisons of taxon richness to various proxies for sampling (e.g. number of fossil-bearing formations or collections [62–66]), completeness metrics that assess preservation bias (e.g. [67–73]) and subsampling approaches (e.g. [58,74–78]). Here we examine the taxon richness of terrestrial lepidosaurs through time, from the Triassic–Palaeogene (252–23 Ma), constructing sampling-corrected taxon-richness curves from an expanded dataset of their occurrences through time. We discuss how solid our basis is for unravelling lepidosaur taxic richness (=‘diversity’) through time and discuss some of the driving factors that might generate these patterns, and note the problems still associated with the lepidosaur fossil record and the fossil record as a whole.

## 2. Material and methods

Triassic–Palaeogene occurrences of lepidosaurs were downloaded from the Palaeobiology Database (PBDB; www.paleobiodb.org), accessed via Fossilworks (www.fossilworks.org) on 27 September 2016, following extensive updating of the taxonomy and occurrences of those taxa already entered (resulting in more than 500 changes to the previously published data) and the addition of approximately 1000 new occurrences after an extensive review of the literature. The relative contributions of different researchers to this dataset are provided in the electronic supplementary material. Data were filtered following download to remove trace fossils, marine taxa and taxonomically indeterminate records, and to correct remaining time bin inconsistencies; the remaining dataset consists of 1971 occurrences representing 449 genera. Genera were used in our analyses because of the problems associated with using species counts in the fossil record (e.g. preservation difficulties preventing identification to species level, uneven taxonomic treatments [79,80]). Of course there are still potential problems with wastebasket taxa even at the generic level, but this is the highest hierarchical level at which we can still study macroevolutionary patterns in detail, as the signal is too coarse at higher taxonomic levels [79]. Using genera allowed us to include specifically indeterminate occurrences, as these comprise a wealth of geographically widespread data (greater than 650 occurrences) that would otherwise be excluded.

In order to account for potential sampling biases in the fossil record, we used Shareholder Quorum Subsampling (SQS; [74,81]) to generate a subsampled richness estimate curve for lepidosaurs, which we compared to the curve of uncorrected face-value genus counts. SQS uses taxon frequencies calculated from the proportion of occurrences that are represented by taxa in each time bin: for example, a taxon that has 15% of occurrences will have a frequency of 0.15 for that bin (its ‘share’). Specimens are drawn and their frequencies are summed to generate a coverage value (the proportion of the total distribution of taxa represented by specimens drawn so far). Sampling stops when a desired level of coverage (the ‘quorum’) is reached. Each species can only have its share counted towards the total once, so a quorum cannot be reached by sampling only multiple specimens of one species. An estimator (Good's *u*) is used to assess the sampling quality of time bins by subtracting the number of single-occurrence taxa from 1. Therefore, an increased number of singletons lowers *u* and is indicative of poor sampling. Good's *u* is used to adjust the quorum of each time bin based on these singleton proportions (to estimate underlying ‘true’ richness) and, therefore, time bins cannot be sampled above their *u* value, and are excluded from analyses where the quorum level is higher than this.

SQS was carried out using a Perl script (v. 4.3; available on request from John Alroy) on global and continental scale data at a quorum level of 0.4, which produces a sufficient measure of relative standing diversity [58,74]. For our analyses, some standard stratigraphic stages were merged to reduce variance in durations between bins (table 1), resulting in interval durations of approximately 9 Myr. Occurrences were only included in our analyses if their stratigraphic ages were known with sufficient certainty to be assigned to a single time bin. With the global data, this excluded 5% of total occurrences, with continental data exclusions ranging from 1–3% (Europe, North America, Indo-Madagascar, Oceania) to 14% (Africa, Asia) and 34% (South America). To highlight underlying data quality, we also marked the number of collections that contributed to each data point in the subsampled curves. In order to avoid problems with Good's *u* for very small sample sizes (it can be overestimated stochastically for very small samples), we also excluded from the figures estimates of richness that were based on data from fewer than five collections.

**Table 1.** Time bins of approximately 9 Myr each used in SQS and GLS analyses, and the standard stratigraphic intervals they correspond to. Previous Triassic bins have no occurrences named at genus level and so are excluded from this table.

bin name	epoch/stage equivalent	base (Ma)	midpoint (Ma)
Pg5	Rupelian–Chattian	33.9	28.465
Pg4	Bartonian–Priabonian	41.2	37.55
Pg3	Lutetian	47.8	44.5
Pg2	Ypresian	56.0	51.9
Pg1	Selandian--Thanetian	61.6	58.8
Pg0	Danian	66.0	63.8
K8	Maastrichtian	72.1	69.05
K7	Campanian	83.6	77.85
K6	Turonian–Coniacian–Santonian	93.9	88.75
K5	Cenomanian	100.5	97.2
K4	Albian	113.0	106.75
K3	Aptian	125.0	119.0
K2	Hauterivian–Barremian	132.9	128.95
K1	Berriasian–Valanginian	145.0	138.95
J6	Kimmeridgian–Tithonian	157.3	151.15
J5	Callovian–Oxfordian	166.1	161.7
J4	Bajocian–Bathonian	170.3	168.2
J3	Toarcian–Aalenian	182.7	176.5
J2	Pliensbachian	190.8	186.75
J1	Hettangian–Sinemurian	201.3	196.05
Tr5	Rhaetian	208.5	204.9
Tr4	Norian	227.0	217.75

As a comparison for the SQS analyses, we also used classical rarefaction (CR), which employs uniform sampling rather than coverage to construct estimated richness curves. Uniform sampling has been criticized for its tendency to dampen genuine diversity signals [74], because the quota is restricted to the level of the bin with the poorest observed taxonomic richness and thus often flattens the ‘corrected’ richness curve. Nevertheless, we compare results obtained from the two subsampling techniques in order to look for common patterns of diversity change through time, as no currently available subsampling method can account for all possible problems with the data. CR results can be found in the electronic supplementary material.

To demonstrate visually the effects of geographical biases on our results, we also plotted the palaeolatitudinal distribution of lepidosaur occurrences through time versus those for other terrestrial tetrapods. Palaeolatitudes are calculated by Fossilworks based on the present-day coordinates of collections using tectonic plate rotation data from Scotese's PALEOMAP Project (www.scotese.com).

We used generalized least-squares regression (GLS) to examine the relationship between uncorrected generic richness, proxies for sampling (terrestrial tetrapod-bearing collections (TBCs) and tetrapod-bearing formations (TBFs)), sea level, non-marine area and palaeotemperature. Time bins remain the same as in the SQS analyses above. GLS allows autoregressive (AR) models to be fitted to the data, which benefits the analysis by reducing the chance of overestimating statistical significance of regression lines due to serial correlation (e.g. [82]). Sampling proxy data was obtained from the PBDB, and non-marine area was obtained from the palaeocoastline reconstructions [83]. For palaeotemperature we used sea surface *δ*^18^O records from Prokoph *et al.* [84] compiled by Mannion *et al.* [77], which we interpolated into 0.1 Ma intervals for ease of subsequent aggregation (see electronic supplementary material); more negative values indicate warmer temperatures and vice versa. All proxy data were then aggregated into within-bin means to enable comparisons with richness data. Sea level [85] and palaeotemperature data are only available from the Jurassic onward, and so analyses including these variables range from bins J4 to Pg5. Duration of time bins was included as an additional non-optional explanatory variable to ensure this was not influencing results, as in [86].

We fitted AR models of orders one or two to combinations of the above explanatory variables, and then compared the results of GLS using Akaike's information criterion for small sample sizes (AICc). Akaike weights were calculated from this information to identify the best combination of explanatory variables from those tested. AIC methods reward models for goodness of fit, but penalize those with higher numbers of explanatory variables [87]. We also manually calculated *R*^2^ values from the GLS output using the generalized *R*^2^ of Nagelkerke [88]. *R*^2^ provides an estimate of the amount of variance in generic richness explained by the variables in each model. To check normality and homoskedasticity of each model's residuals, we used the Jarque–Bera [89] and Breusch–Pagan [90] tests. The sampling proxies and generic richness were ln-transformed prior to analysis to ensure normality and homoskedasticity of residuals. All analyses were carried out in R v. 3.4.3, using the packages lmtest v. 0.9–35 [91], nlme v. 3.1–131 [92], qpcR v. 1.4–0 [93] and tseries v. 0.10–40 [94], following the approach of Benson & Mannion [95].

## 3. Results

### 3.1. Subsampling

**Figure 1.**
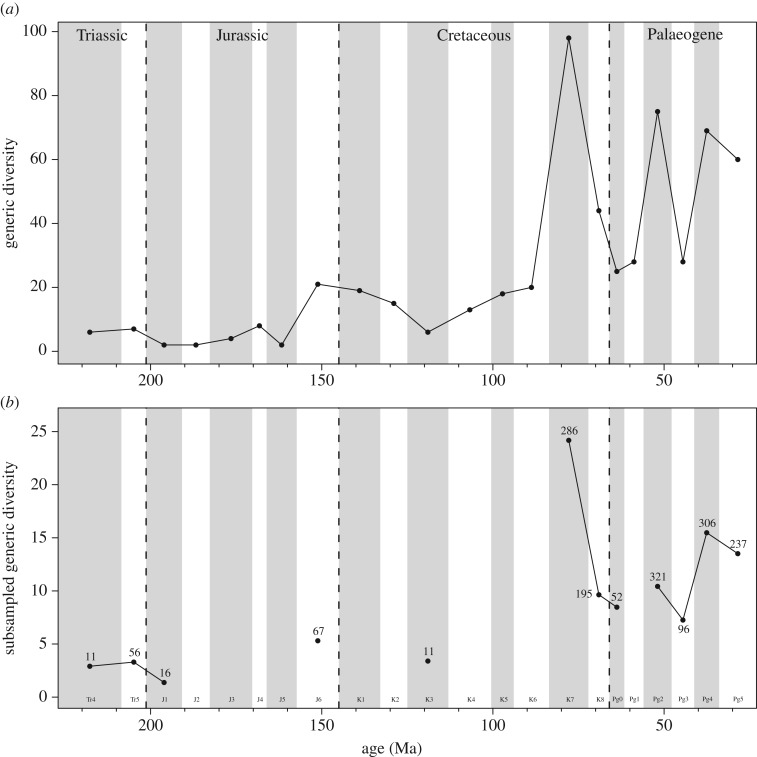
Terrestrial lepidosaur global generic diversity from Triassic–Palaeogene using (*a*) uncorrected (raw) diversity data and (*b*) subsampled (SQS) diversity data at quorum 0.4. Numbers on (*b*) indicate number of collections drawn for each time bin subsampled, as an indicator of underlying data quality. Time bin durations are explained in table 1.

Patterns of relative change in subsampled richness estimates for pooled global occurrences are highly similar to face-value genus counts (figure 1). Many time bins do not provide subsampled richness estimates, as they are too poorly sampled to meet the lowest quorum level (indicated by low values of Good's *u*). Even at lower quorum levels (see electronic supplementary material, figure S1 for quorum levels 0.3–0.6) the situation is not much improved, and a signal could not be recovered for many Jurassic and mid-Cretaceous time bins.

There is a slightly higher generic richness before the Triassic–Jurassic (Tr–J) boundary than afterwards in both the face-value and corrected data. At a quorum level of 0.4 there are few well-supported data points prior to the Late Cretaceous. The Late Cretaceous sees the highest ‘global’ standing diversity estimate, in bin K7 (Campanian), followed by lower estimated richness in K8 (Maastrichtian) and across the K–Pg boundary. There is a subsequent gap in coverage until Pg4 (Bartonian–Priabonian) in the 0.4 quorum data, but at a quorum level of 0.3 (electronic supplementary material, figure S1). The data suggest that the trend is more like that seen in the face-value data, with sequential peaks and troughs culminating in a lower diversity estimate in the Oligocene than in the preceding time bin (Pg4–Pg5), which is still relatively high compared with the overall curve. Very similar trends are recovered using CR (electronic supplementary material, figure S2), though there are some issues with missing data and flattened trends at lower quotas (see Discussion). Global curves are of limited use when interpreting how richness estimates change through time, however, given the unequal input of individual regions due to geographical sampling biases (see below). Consequently, we focus on regional trends herein. However, as many previous studies have tended to focus on worldwide trends, we provide the global curve for comparison (figure 1), in order to demonstrate how it is influenced by smaller-scale fluctuations in richness and to provide a pictorial summary of the current state of the lepidosaur fossil record.

**Figure 2.**
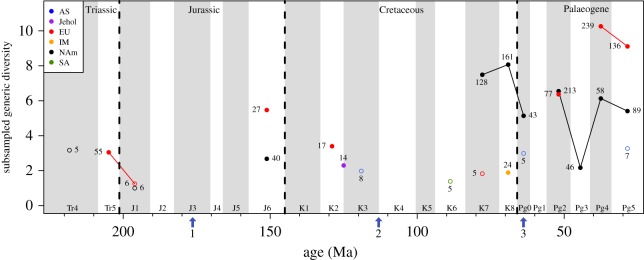
Subsampled terrestrial lepidosaur generic diversity from Triassic–Palaeogene at quorum 0.4, for individual continents: AS, Asia; EU, Europe; IM, Indo-Madagascar, NAm, North America; SA, South America. Also included is the combined Jehol Group. Numbers indicate number of collections drawn for each time bin subsampled, as an indicator of underlying data quality. Arrows indicate approximate timings of major finds in the lepidosaur record: 1, earliest squamate fossils; 2, earliest snake fossils; 3, earliest amphisbaenian fossils. Time bin durations are explained in table 1.

Splitting the ‘global’ data into continental regions reveals a major problem of uneven sampling between continents (figure 2) and demonstrates that literal readings of the apparently ‘global’ curve can lead to the recovery of erroneous macroevolutionary signals. This result derives from the fact that at some times the entire ‘global’ signal is estimated almost entirely from a single productive continent, while other undersampled regions contribute little or no data to the ‘global’ picture. For example, Indo-Madagascar has exceptionally poor coverage, and robust SQS results (i.e. those underpinned by more than five collections per time bin, see above) are only recovered in one time bin. Africa and Oceania have so few collections providing data for each time bin that no robust results were obtained at all and these areas were excluded from figure 2 (see electronic supplementary material, figure S6). Therefore, little can be said about diversity trends on these continents except that in the Maastrichtian (K8), richness in Indo-Madagascar is much lower than in North America. There are further substantial differences in the numbers of collections attributed to each data point between continents, which are particularly marked between the relatively well-sampled areas of North America and Europe versus the rest of the world. A drop in diversity was recovered across the Tr–J boundary in Europe, but other regions do not reach the minimum quorum level in the final Triassic bin, so it is unclear what is occurring outside Europe at this time. This means that the ‘global’ record from this time is almost entirely driven by the European signal. Comparatively high diversity was found in the Late Jurassic (K6; Kimmeridgian–Tithonian) in Europe and becomes lower in the Early Cretaceous (K2), but after this time a robust generic richness signal is absent until the Palaeogene, and it is not possible to track sequential trends in richness before this time. Asia and South America have a relatively intermediate to low estimated richness during the Cretaceous, but the data available are sparse and so these estimates are less reliable. We also plotted the estimated generic richness of the amalgamated Jehol Group, which crosses several of our time bins and was thus excluded from our other analyses, in order to show the generic richness in this Lagerstätte. The richness estimate is relatively low, but is more robustly supported than the Asian K3 point.

In the latest Late Cretaceous, North America shows the same proportionately high levels of generic diversity as the ‘global’ curves, but with one notable difference. In North America, K8 has a slightly higher diversity than K7 (probably within stochastic error of each other) and the drop across the K–Pg boundary is steeper than in the ‘global’ curve. In addition, this latest Cretaceous peak no longer represents the highest diversity in our time series, and it is proportionately much lower than the peak seen in the ‘global’ curve at this time (which is due to the fact that the ‘global’ curve is an amalgamation). No other continents exhibit any sequential trends through this time period, but seem to have a low to intermediate diversity when compared to North America. After a break, we see the same peak and trough pattern as in figure 1 for Pg2–5 in North America, and partially in the European data (the missing trough is present at quorum 0.3; see electronic supplementary material, figure S4), although it is more pronounced than in the ‘global’ data. In Pg4 there is extremely high relative richness in Europe, and high diversity in North America, followed by decreases in both regions over the Eocene–Oligocene boundary, as in the ‘global’ data.

**Figure 3.**
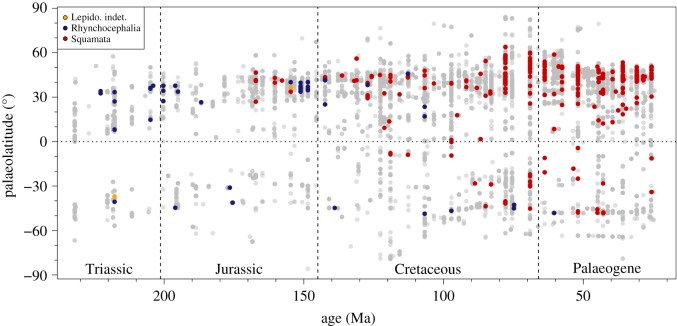
Palaeolatitudinal distribution of terrestrial lepidosaur occurrences from Triassic–Palaeogene; light grey circles indicate non-lepidosaur tetrapod occurrences. ‘Lepido. indet’, lepidosaur taxa of basal or unknown placement.

The disparity between sampling in different continents is also highlighted by the pattern shown in figure 3, in which the palaeolatitudes of every terrestrial lepidosaur occurrence through time are plotted against those for all other terrestrial tetrapods. It is clear that the vast majority of lepidosaur occurrences are from the Northern Hemisphere, and this appears to be a trend for tetrapods as a whole. The absence of lepidosaur specimens at very high latitudes is notable, even when other tetrapods are present.

To test sensitivity to taxonomic sampling, we separated snake occurrences from the dataset and ran new analyses on these ‘snake-only’ and ‘other lepidosaur’ datasets. While snakes are too poorly sampled to produce meaningful results, with the exception of a slight drop across the Eocene–Oligocene boundary, their exclusion from the lepidosaur dataset causes a few points that had previously dropped out of analyses (Pg1 of North America and Pg3 of Europe) to be recovered (see electronic supplementary material, figure S7). This is most likely due to a decrease in the number of singleton taxa that would have otherwise been included in these bins, thus raising the maximum quorum level that they could be sampled at, allowing us to recover a signal at quorum 0.4.

### 3.2. Multivariate analyses

We examined various explanatory variables that may be driving face-value generic diversity using GLS (table 2). Duration was not found to be significant for any of the models tested and did not change the order of the best models, so these results are presented in electronic supplementary material appendix S2. Seven models had higher AICc weights than the null model. The best models based on weights all featured TBCs, except for the TBFs model which was selected as the fifth best. The TBCs + *δ*^18^O palaeotemperature model emerged as the best model (with a weight of 0.72), followed by TBCs only (0.16) and then TBCs + non-marine area (0.07). While TBCs + palaeotemperature has been identified as the best model, with a moderately good *R*^2^ value, within this model TBCs is highly significant (*p* < 0.0001) but the palaeotemperature proxy is not (*p* = 0.76). In all of the best model combinations, TBCs is the only variable with significant *p*-values. Furthermore, the AICc values of this model and the TBCs-only model are very similar, so the AICc weight of the TBCs-only model is low but non-negligible. This makes it more difficult to choose between these two models, but implies strongly that sampling (TBCs) is the most important explanatory variable.

**Table 2.** Summary of various model fits to observed (raw) generic diversity of lepidosaurs. *N* = 17 time bins; ‘TBCs’, tetrapod-bearing collections; ‘TBFs’, tetrapod-bearing formations; ‘*δ*18O’ is our palaeotemperature proxy. Columns show coefficients of explanatory variables within each model (columns 2–13), and then overall model fit parameters (columns 14–17). Best three models as chosen by AICc are bolded for ease of reference.

	sampling (TBCs or TBFs)			sea level (SL)			non-marine area (NMA)			δ^18^O						
	slope	*t*-value	*p*-value	slope	*t*-value	*p*-value	slope	*t*-value	*p*-value	slope	*t*-value	*p*-value	*R*^2^	log-likelihood	AICc	AICc weight
null model	—	—	—	—	—	—	—	—	—	—	—	—	—	−21.3	48.9	<0.001
TBFs	1.216	2.96	0.0097	—	—	—	—	—	—	—	—	—	0.310	−18.2	45.2	0.004
**TBCs**	**1**.**018**	**5**.**86**	**<0**.**0001**	—	—	—	—	—	—	—	—	—	**0**.**559**	−**14**.**3**	**37**.**5**	**0**.**163**
SL	—	—	—	0.025	4.27	<0.0001	—	—	—	—	—	—	−0.137	−22.4	55.6	<0.001
NMA	—	—	—	—	—	—	−0.041	−1.97	0.0678	—	—	—	−0.110	−22.2	55.2	<0.001
*δ*^18^–O	—	—	—	—	—	—	—	—	—	−0.029	−0.10	0.9248	0.017	−21.2	53.2	<0.001
SL + NMA	—	—	—	0.025	4.08	0.0011	0.013	0.78	0.4496	—	—	—	−0.599	−25.3	64.4	<0.001
*δ*^18^O + SL	—	—	—	0.027	4.68	0.0004	—	—	—	0.244	1.31	0.2117	−0.126	−22.3	58.5	<0.001
*δ*^18^O + NMA	—	—	—	—	—	—	−0.019	−0.66	0.5228	0.043	0.13	0.8958	−0.376	−24.0	59.9	<0.001
TBFs + SL	0.960	2.37	0.0326	0.015	2.26	0.0406	—	—	—	—	—	—	0.150	−19.9	53.7	<0.001
TBFs + NMA	1.528	4.06	0.0012	—	—	—	0.022	1.23	0.2378	—	—	—	0.090	−20.5	54.8	<0.001
TBFs + *δ*^18^O	1.365	3.38	0.0045	—	—	—	—	—	—	0.060	0.27	0.7918	0.279	−18.5	50.9	<0.001
TBFs + SL + NMA	1.030	2.56	0.0238	0.015	2.22	0.0450	0.020	1.38	0.1909	—	—	—	−0.144	−22.4	62.2	<0.001
TBFs + SL + *δ*^18^O	0.916	2.35	0.0350	0.017	2.73	0.0171	—	—	—	0.225	1.41	0.181	0.151	−19.9	57.2	<0.001
TBFs + NMA + *δ*^18^O	1.533	3.93	0.0017	—	—	—	0.026	1.13	0.2791	−0.085	−0.33	0.7468	0.046	−20.9	59.1	<0.001
TBFs + SL + NMA + *δ*^18^O	0.967	2.26	0.0433	0.016	2.23	0.0452	0.009	0.44	0.6660	0.140	0.56	0.5849	−0.197	−22.8	67.1	<0.001
TBCs + SL	1.171	6.99	<0.0001	0.000	0.08	0.9357	—	—	—	—	—	—	0.611	−13.3	40.4	0.039
**TBCs + NMA**	**1**.**176**	**14**.**11**	**<0**.**0001**	—	—	—	**0**.**004**	**0**.**71**	**0**.**4873**	—	—	—	**0**.**638**	**−12**.**7**	**39**.**2**	**0**.**072**
**TBCs + δ^18^O**	**1**.**183**	**14**.**13**	**<0**.**0001**	—	—	—	—	—	—	**0**.**022**	**0**.**31**	**0**.**7576**	**0**.**724**	−**10**.**4**	**34**.**6**	**0**.**716**
TBCs + SL + NMA	1.152	6.61	<0.0001	0.001	0.16	0.8771	0.004	0.70	0.497	—	—	—	0.380	−17.2	51.8	<0.001
TBCs + SL + *δ*^18^O	1.142	6.05	<0.0001	0.001	0.24	0.8107	—	—	—	0.031	0.37	0.7198	0.534	−14.8	46.9	0.002
TBCs + NMA + *δ*^18^O	1.172	13.23	<0.0001	—	—	—	0.007	0.72	0.4831	−0.044	−0.38	0.713	0.584	−13.8	45.0	0.004
TBCs + SL + NMA + *δ*^18^O	1.181	5.75	0.0001	0.000	−0.05	0.9645	0.007	0.64	0.532	−0.049	−0.33	0.7504	0.308	−18.2	57.8	<0.001

## 4. Discussion

### 4.1. Lepidosaur diversity through time

SQS and CR recovered the same broad diversity trends through time, but the CR quota levels that recover sufficient data are small and missing more points than the SQS analyses (figures 1*b* and 2; electronic supplementary material, figures S2 and S3). Unfortunately, CR curves suffer from the problem of dampening at lower quota levels (see [74] for a full discussion of this) and this means that some quotas with useful information exhibit very shallow fluctuations in diversity. As a result, we discuss only the SQS results here, but full CR results are available in the electronic supplementary material.

The lepidosaur fossil record is variably sampled, but sampling is often poor across multiple time bins and regions, as seen in figures 1*b* and 2 versus figure 1*a*, where many time bins dropped out of the SQS analysis as the data could not be subsampled at even very low quorum levels. This issue obscures overall diversity trends through time and prevents us from presenting robust scenarios for a large portion of early lepidosaur history. Similarly, sampling affects what we observe in ‘global’ trends; many portions of the sampling-corrected ‘global’ curve result from a small number of continents producing most of the signal (figure 2). Additionally, ‘global’ curves tend to be cumulative based on the number of continents contributing to a time bin (i.e. double the continents, double the estimated richness) in absence of any underlying changes in diversity. For example, Benson *et al*. [58] found that the palaeogeographic spread of global fossil localities explained 72% of the variance in subsampled ‘global’ species-richness for terrestrial tetrapods (and see [74,96]). This means that apparently ‘global’ richness curves tell us predominantly about the number of continents that have been sampled for fossils in each interval and should be approached with caution.

North America and Europe are well sampled compared with the rest of the world, which affects what we can conclude about overall trends in taxic richness. This Northern Hemisphere bias is shown clearly in the palaeolatitudinal distribution of occurrences through time (figure 3), which is similar to that recorded for other tetrapod groups [77,78]. This bias appears to be much more acute for lepidosaurs, but it is not currently possible to determine if it reflects sampling issues that might be specific to lepidosaurs (such as their small size and low recovery potential), a genuinely different geographical distribution from that of other tetrapods (due to ecological or physiological dissimilarities) or a combination of these factors.

Despite this, the signal that we recover is still informative for first-order patterns of lepidosaur diversity. At a quorum level of 0.4 (and up to quorum 0.6; see electronic supplementary material, figure S1), there is a stable and low level of rhynchocephalian diversity in the latest Triassic that declines across the Tr–J boundary. This interval is only resolved for Europe (as all other continents are too poorly sampled; figure 2) and studies of taxon changes in the fissure-fill faunas of the southwest of England indicate a shift from a Late Triassic fauna dominated by reptiles, with a diverse array of rhynchocephalians, to a mammaliaform-dominated Early Jurassic fauna (we have no evidence to discern whether this is a local environmental signal or more widespread) [97]. Early Jurassic rhynchocephalians are reduced to two genera (*Gephyrosaurus* and *Clevosaurus*), though they remain dominant in abundance despite low generic diversity [97]. By contrast, the Tr–J boundary effect on mammal generic diversity is negligible [98]. This faunal shift might be related to the end-Triassic mass extinction, which was probably caused by environmental change associated with long-term volcanism in the Central Atlantic Magmatic Province (CAMP [99–101]).

There is a sampling gap (caused by poor data), which prevents us from estimating richness for many regions and intervals, through most of the Jurassic and Cretaceous until the latest Cretaceous. It is punctuated by a few individual points in various time bins where the presence of various Lagerstätten (areas of exceptional quality or quantity of preservation) have ensured that sampling is good enough to be detected at this quorum level. These include the Late Jurassic (J6) localities of the Morrison Formation (western North America), Solnhofen (Germany), Cerin (France) and Guimarota (Portugal), and the Early Cretaceous sites of Las Hoyas (K2; Spain) and the Jehol Group (partially in K3; China). This is unfortunate, as we lack information on how diversity patterns were affected by the first recorded occurrences of squamates in the Middle Jurassic [20,22,23] and by the diversifications of the many extant terrestrial clades that are thought to have occurred during the Early Cretaceous (e.g. scincids, xenosaurid anguimorphs, acrodont iguanians [102,103]). At a quorum level of 0.3 (electronic supplementary material, figure S1), we recover a rise in diversity across the J–K boundary in the ‘global’ subsampled curve, contrary to the results seen in the face-value generic curve, and to those obtained for the majority of large-bodied contemporaneous reptiles [77,104], but that is consistent with the findings of other studies on mammals [98], non-marine turtles [78] and small-bodied tetrapods, in general [104]. However, the ‘global’ curve here is composed almost entirely of the European record (electronic supplementary material, figure S4), so this should be interpreted as a regional phenomenon.

Southern Hemisphere (Gondwanan) continents are represented in time bin K6, but there are too few localities to be interpretable. Rhynchocephalians still occupy South America at this time, despite their disappearance from Laurasian continents during the Jurassic and Early Cretaceous [28,105], and are found alongside numerous snakes (which are uncommon in Laurasia at this time [28]).

In the ‘global’ data, we recover the highest diversity for our entire dataset in the Campanian (K7). However, in the continental-level curves this large Late Cretaceous peak disappears (as it is an agglomeration of varying regions) and in the North American curve, the difference between these bins and the Palaeogene is not as large; in Europe, it appears as if the Eocene has the largest richness (Pg4), but there is only one poorly supported Late Cretaceous point (and fewer points overall) so comparisons are more difficult. This is important to note because, as mentioned above, apparently ‘global’ patterns are unreliable. For example, the presence/absence of data from different regions in adjacent time intervals has the potential to create false peaks and troughs that could lead to misinterpretations of true ‘global’ richness.

Only North America is well sampled enough to show sequential diversity during the end-Cretaceous, with a wealth of squamate taxa from Canada and the USA that increases slightly in richness from the Campanian to the Maastrichtian (however, these estimates are probably within stochastic error of each other; at higher quorum levels they are nearly or exactly the same; see electronic supplementary material, figure S5). This result is contrary to that of Nydam [50], who found higher taxonomic richness in the Campanian than in the Maastrichtian, but noted that this result could have been affected by sampling biases, as he did not use any sampling-correction methods. Lower relative richness is found in Indo-Madagascar at this time (and also in K7 Europe, but this data point is not as robustly supported, with only five collections). ‘Global’ Campanian high diversity is heavily influenced by finds from Mongolia, which has an incredibly rich fauna (with greater than 50 genera), in conjunction with North America. Unfortunately, this Asian signal is lost when we examine diversity at continent level, as there are numerous single-occurrence taxa in these Mongolian faunas and so the SQS algorithm deems the continent too poorly sampled to be counted. Moreover, it is likely that revision of the Mongolian faunas is needed, as there are many named taxa (e.g. [106]) that have not been examined in detail subsequently and the generic richness of these sites might change substantially in the future. This observation highlights a major issue in diversity studies in palaeontology as a whole, which is reliance on an existing body of literature that is heavily biased towards the description of new taxa or localities, rather than documenting in detail every occurrence of common or widespread taxa. For example, museum collections that house large numbers of duplicate specimens from places like the Campanian of China and Mongolia (e.g. the Institute of Vertebrate Paleontology and Paleoanthropology, Beijing; S.E.E. 2017, personal observation) that are not individually listed in the literature and so do not contribute towards publications-based analyses like these. However, these collections data can be made available and will be potentially important for future work, through increasing the known occurrences of taxa currently published as singletons, for example.

The K–Pg boundary is heavily sampled due to its macroevolutionary importance and many tetrapod lineages went extinct at this time [107]. Lepidosaurs are no exception to this, as the large-bodied polyglyphanodontians (also known as borioteiioids) died out and caused a drop in ‘global’ diversity across the boundary [108]; Wilson [109] found the same drop for larger-bodied mammals. This is observable in the North American data, as polyglyphanodontians dominated assemblages for the entire Late Cretaceous, and there is a good Danian record in the region to observe faunal changes across the boundary. This is consistent with the findings of [108], who observed a significant loss of Cretaceous-occurring genera across the boundary. Faunal recovery is rapid, however, and by the end of the Danian a more modern squamate fauna is beginning to diversify. This can be seen in the continent-level curves at a quorum level of 0.3 (electronic supplementary material, figure S4), but North America is not well sampled enough in the later Paleocene for a sequential trend to be detected at a quorum of 0.4. The same is true of the other continents even at the lower quorum level, and so we cannot draw many conclusions regarding the effects of the K–Pg extinction on lepidosaur richness and recovery.

After the sampling gap in the early Paleocene, we reach a period of excellent sampling in both North America and Europe that reveals some interesting patterns. The Paleocene–Eocene Thermal Maximum (PETM) heralded a much warmer climate and opportunities for taxa to diversify into niches and areas that may have been thermally limiting to smaller ectothermic taxa previously [110–112]. This was followed quickly by the Early Eocene Climatic Optimum (EECO; e.g. [113]). At this time, diversity in Europe is much higher than in the Campanian, with a multitude of localities yielding rich faunas of snakes and lizards. Iguanians appear for the first time in Europe [49] and amphisbaenians were numerous by this time, having diversified at least as early as the Paleocene [30,51], and are found in most European localities despite their fossorial nature. Diversity is at approximately the same level in North America, with many Paleocene lineages persisting and diversity boosted by probable immigration from lineages that were previously restricted to more southerly environments (e.g. stem-polychrotid iguanians [114]).

A large drop in diversity is seen in North America during Pg3 (Lutetian) and is mirrored in Europe at a quorum level of 0.3 and also at a quorum of 0.4 when snakes are excluded from the analyses (see electronic supplementary material, figure S4 and S7). This may be linked to the end of the EECO, where temperatures begin to decline [113]. In Europe, much of the middle Eocene is poorly sampled due to a lack of localities compared with the early Eocene. Some Lagerstätten occur at this time (Messel and Geiseltal, Germany), but it is difficult to compare fossils from these localities to those in other time bins due to extreme differences in preservation [51]. Interestingly, amphisbaenians are not found even in localities with the special taphonomic conditions of Lagerstätten, suggesting a genuine absence (or very low diversity) at this time [35] or, more likely, a preservation bias against fossorial animals. This low richness differs from the findings of Mannion *et al*. [77] for crocodylians, where European generic richness was relatively high in the middle Eocene, with a much lower richness in the late Eocene. This contrasts with our prior expectation that key groups of endotherms would show similar patterns of change in genus richness, coordinated by environmental change. This is also true of their North American data points, which are the inverse of what we observed for bins Pg2–4, with high richness in Pg3 and lower richness on either side of this bin.

**Figure 4.**
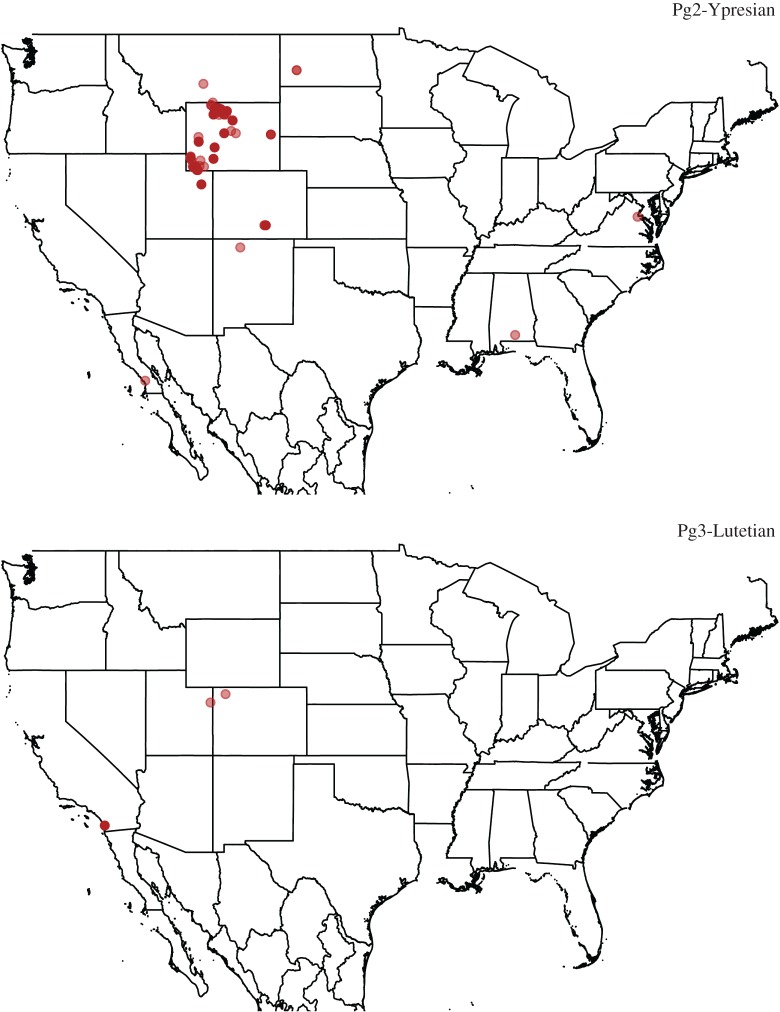
Distribution of North American lepidosaur localities in bins Pg2 and Pg3. Darker shades indicate layered localities.

There is a lack of squamate localities in central North America from the middle Eocene [112], which makes it difficult to track sequential faunal changes in this area. Woodburne *et al*. [115] found a loss in diversity for mammals after the EECO, although these data are uncorrected for sampling, and an associated loss of floral diversity that was connected with increasing aridity during this period. The same may have been true for lizard genera, particularly for the more thermophilic taxa that supposedly immigrated into the area during the EECO [114], but studies of early and late Eocene localities in Wyoming show that many of these taxa seem to have persisted throughout the entire period [112]. Squamates, therefore, do not appear to be as affected as mammals (at least in central North America), but the middle Eocene sampling gap makes it difficult to tell if diversity here was consistent, or if taxa migrated away and then returned during climatic amelioration in the latest Eocene [113]. The large drop in recovered richness from Pg2 to Pg3 is almost certainly an artefact of sampling entirely different areas of North America during these two time bins, which SQS cannot account for (figure 4). The early Eocene record comprised localities mostly from Wyoming, whereas the middle Eocene squamate localities are Californian. It may be that diversity was always proportionately lower in California than in Wyoming, but without good coeval data from both regions it is currently impossible to tell.

Both Europe and North America have high generic diversity in the late Eocene, in contrast to the lows in the previous interval. Diversity in Europe is at its maximum during this period, with an incredible array of snakes, lizards and amphisbaenians from a wide range of localities. The rich fauna is composed of taxa that were already established in the region in combination with the first European appearance of cordyliforms, the latter being indicative of an environment that was still warm enough to support thermophilic taxa despite the overall cooling during the Eocene [116]. Amphisbaenians continued to diversify, and the first blanid (an extant taxon endemic to Europe) appears in France at this time [35]. A decline in small-bodied mammalian diversity at this time might have allowed niche diversification for squamate taxa [45] and been a contributing factor underpinning this high diversity. Late Eocene faunal richness in central North America (Wyoming) is very similar to that of the early Eocene, meaning that (central) North American squamates also continued to flourish.

The Eocene–Oligocene (E–O) boundary is marked by a sudden substantial drop in temperature, which caused increased seasonality and the onset of glaciation in Antarctica [113,117,118], and is associated with a significant extinction event known as the Grande Coupure. The extinction was first recognized in the terrestrial realm for mammals [119–123], but has been recognized subsequently for squamates (e.g. [51,124,125]) and pseudosuchians [77]. The event is believed to have been much more severe in Europe, as (combined with climate change) a major fall in eustatic sea level [85,126] connected a long-isolated Europe with Asia at this time, allowing Asian taxa to immigrate into Europe and outcompete established taxa [127]. Squamate turnover may also have been exacerbated by the diversification of mammalian carnivorans [125]. Our data show a fall in diversity across the boundary (Pg4–5) in both Europe and North America, which is slightly steeper in Europe, but diversity remains relatively high afterwards when compared with that across the entire time span of our study.

In Europe, it is only possible to track the Grande Coupure in detail for squamates sequentially in the Quercy Phosphorites localities of southern France (although some unpublished work also exists for the Hampshire Basin of the UK [128]), which limits our understanding of when certain taxa disappeared from the continent as a whole. What is certain, however, is that Oligocene faunas are markedly different from late Eocene faunas with the exception of a small handful of survivors that did not persist for long after the boundary (except *Plesiolacerta* [51]). Glyptosaurine lizards disappear from Europe entirely and the modern snake family Colubridae makes its first appearance. Iguanians, once thought to have gone extinct in Europe at the E–O boundary, appear in one locality in the late Oligocene of France (*Geiseltaliellus*; [129]). Intense sampling of other Oligocene French localities has yet to produce further specimens, suggesting a genuine absence during the early Oligocene, but it is impossible to tell if they retreated to refugia in other parts of Europe due to the scarcity of localities in other regions. It is difficult to track the progress of Asian immigration also, as the number of eastern European and Asian localities from this time bin are limited. Diversity remains relatively high in our curve after the E–O boundary, which probably represents the rapid re-diversification of taxa (and the possible immigration of Asian taxa, as occurred for mammals) by the end Oligocene, filling vacant niches [45,125].

In North America, the Grande Coupure event was previously thought to be negligible for lepidosaurs, but new finds in the late Eocene suggest that North American squamates also experienced a similar but less severe extinction at the end of the Eocene [130]. This is consistent with what we find in our sampling-corrected curves, though the decline is not marked. The available data points may be within stochastic error of each other, as between the K7 and K8 bins, although the curve at quorum level 0.5 still shows a similar decrease (unlike with K7–8 where the trend becomes shallower; see electronic supplementary material, figure S5). Many taxa with tropical affinities disappeared, but it is impossible to tell whether they migrated southwards or went extinct due to a lack of coeval localities from tropical North America [112]. North American mammal taxa at this time apparently passed through the boundary relatively unscathed [131], possibly due to the fact that mountain uplifting in the middle–late Eocene pre-adapted taxa to cooler climatic conditions [121].

We recovered a rise across the Eocene–Oligocene boundary for taxa in Asia, but due to a very low number of localities the data are not adequate to support robust patterns and so were excluded (see electronic supplementary material, figure S5). Previous studies of central Asian mammals suggest that they experienced a similar turnover to those in Europe and North America, with the transition from a warm temperate to an arid or semi-arid climate causing a change in the dominant faunas from large-bodied perissodactyls to smaller rodents and lagomorphs [123,132]. Less is known about squamate faunas over the boundary in this region, although it has been suggested that early Oligocene herpetofaunas featured many taxa analogous to modern arid-dwelling species, in comparison to late Eocene faunas that were more indicative of humid climatic conditions and mild winter temperatures [44].

It is not possible to examine the effects of the Grande Coupure on squamate taxa from the other continents: low diversity in Africa and Oceania is recovered in our analyses before and after the E–O boundary, respectively (electronic supplementary material, figure S5), but these data are too incomplete to reveal sequential patterns in taxon richness.

It is important to note that many first occurrences of major lepidosaur taxa discussed above conflict greatly with the divergence dates derived from molecular phylogenies (e.g. [103,133,134]). These phylogenies, while disagreeing on smaller-scale details, propose that crown group squamates began to diversify as early as the Middle Jurassic [133], with amphisbaenians diversifying approximately 100 Ma (mid-Cretaceous [133]) or as early as 146 Ma (latest Jurassic [103]). Snake phylogenies appear to provide better divergence estimates, with some studies placing their divergence date at approximately 125–130 Ma (Early Cretaceous [103]), which is almost coincident with the earliest unequivocal snake fossil finds. Total evidence dating [134], which attempts to reconcile molecular and morphological characters, comes closer to the divergence dates provided by the fossil record (e.g. diversification of Amphisbaenia at approx. 60 Ma), but still proposes large ghost lineages for other groups due to the lack of pre-Late Cretaceous material to calibrate these models. It is difficult to test these and other molecular-based estimates of diversification times and rates when the fossil record is so poor. So, at least for now, the origination times of major taxa remain unresolved.

### 4.2. Multivariate analyses

As mentioned above, it is difficult to select the best model from our results, as AICc values for the best models are very similar. With regard to AICc weights, the TBCs + *δ*^18^O palaeotemperature model is four times better than the TBCs-only model. This is not a large difference compared to, for example, the difference between these two models and the null model (where palaeodiversity is constant and variation is explained by error), which has a weight of less than 0.001. While the *R*^2^ value is improved by the addition of these extra explanatory variables (but still remains moderate at 0.72 in the best model), the non-significant *p-*values of every explanatory variable besides TBCs in all of the best models suggests that the sampling proxy (number of collections) is the important factor in our results. This finding is consistent with research on pterosaurs [65], marine reptiles [56], sauropodomorph dinosaurs [95], Late Cretaceous turtles [135] and dinosaurs as a whole [75], which also found the number of collections as either the best explanatory variable, or the best in conjunction with non-marine area and/or the presence of Lagerstätten (which is another proxy for sampling biases). The low to intermediate *R*^2^ values of all the best models (0.72 or lower; table 2), however, demonstrate that there is a good proportion of variance in the face-value generic data that remains unexplained by sampling alone. This suggests that other factors were also driving lepidosaur diversity through time, perhaps biotic interactions that are difficult to test for in the fossil record, such as competitive exclusion or ecological responses to local environmental change, or other abiotic variables or evolutionary innovations that were not tested in this study.

## 5. Conclusion

Overall, we recover some interesting diversity trends from the Mesozoic–Palaeogene lepidosaur fossil record, which have served to test the observations made by others while correcting for previously overlooked unequal sampling issues. Lepidosaur richness was low from the Triassic until the Late Cretaceous, with a few exceptions (primarily the Late Jurassic of Europe, which features numerous localities with exceptional preservation, such as Cerin, Solnhofen and Guimarota). Richness became higher in the Late Cretaceous (Campanian–Maastrichtian), in North America, but was low in Indo-Madagascar. North America is well sampled enough to observe a decrease in richness across the K–Pg boundary (which saw a loss of larger-bodied taxa during the mass extinction) that remained at a moderately high level compared to earlier intervals.

In the Palaeogene, both North America and Europe maintained a high level of richness that fluctuated with climate, except during the Lutetian (probably due to the limited geographical spread of localities in North America; Europe is too poorly sampled at quorum 0.4). In Europe, richness was much higher at the end of the Palaeogene than every other interval recovered in the analysis. There was a fall in richness in both continents following the Grande Coupure at the Eocene–Oligocene boundary, though richness remained moderately high in North America and very high in Europe moving towards the Neogene. This implies a relatively late acquisition of the incredible lepidosaur richness that we observe today, though we are lacking much of the data that would help us understand how they became so diverse, particularly in the tropics.

Unfortunately, the lepidosaur record suffers from many issues related to sampling biases, particularly geographical biases, which obscure sequential patterns in diversity. Rhynchocephalians disappear from the Northern Hemisphere in the Early Cretaceous, and their record (and the Southern Hemisphere record) is so poor post-Cretaceous that we know little about the processes that restricted them to the limited distribution they occupy today. It is also impossible to pinpoint the geographical origins of lizards or snakes, aside from inferences based on their relative early abundances in Laurasia and Gondwana, respectively [27], as Jurassic and Early Cretaceous records are currently too poor for most continents. Even in time bins with relatively good sampling there are periods when we have locally excellent records that cover only a small proportion of the total land area available (e.g. Western Europe, Western Interior of North America). Although it is tempting to extrapolate the signals generated from these unevenly distributed data to large-scale macroevolutionary patterns, these results demonstrate that it is crucial to examine these data at regional and local levels to avoid generating false hypotheses. However, until more data become available it behoves us to refine the signals we can extract from the data we have available in order to try and reconstruct rigorously tested patterns of past diversity, as these form useful benchmarks for understanding the origin and evolution of the modern biota. In particular, we must continue to look for new fossiliferous localities worldwide, particularly in the Southern Hemisphere, in order to improve our data and the robustness of our conclusions.

## Supplementary Material

Additional figures and tables

## Supplementary Material

PBDB and GLS data

## Supplementary Material

Code for removing unneeded taxa from PBDB data

## Data accessibility

The datasets and additional figures supporting this article have been uploaded as part of the electronic supplementary material.
