# Supplementary material for: Lepidosaurian diversity in the Mesozoic–Palaeogene: the potential roles of sampling biases and environmental drivers
Source: R Soc Open Sci. 2018 Mar 21;5(3):171830. doi: 10.1098/rsos.171830 (PMC5882712; doi:10.1098/rsos.171830)
Supplement: Additional figures and tables [file rsos171830supp1.pdf]

Lepidosaurian diversity in the Mesozoic–Paleogene: the potential roles of sampling biases and environmental drivers

T.J. Cleary, R.B.J. Benson, S.E. Evans and P.M. Barrett

SI Appendix 1: supplementary figures and tables

Contents

SI Table 1: List of included countries in each continental area

SI Figure 1: SQS quorum levels 0.3-0.7

SI Figure 2: Classical rarefaction quota levels 15, 30 and 45 (global results)

SI Figure 3: Classical rarefaction quota levels 15 and 30 for individual continents

SI Figure 4: SQS quorum level 0.3 for individual continents

SI Figure 5: SQS quorum levels 0.5 and 0.6 for individual continents

SI Figure 6: SQS quorum level 0.4 including points with very low collection numbers

SI Figure 7: SQS quorum level 0.4 for snakes and non-snake lepidosaurs

SI Table 1: List of continent assignments of the countries in our dataset. Russia is included twice, and the data was edited to reflect this, in order to separate out the localities closer to Europe or Asia.

**Continent**	**Included countries**
Africa	Algeria, Egypt, Libya, Morocco, Niger, South Africa, Sudan, Tanzania
Asia	China, Japan, Kazakhstan, Kyrgyzstan, Lebanon, Mongolia, Myanmar, Pakistan, Russian Federation, South Korea, Uzbekistan
Europe	Austria, Belgium, France, Germany, Germany, Hungary, Italy, Luxembourg, Poland, Portugal, Romania, Russian Federation EU, Spain, Switzerland, United Kingdom
Indo-Madagascar	India, Madagascar
North America	United States, Canada, Mexico
Oceania	Australia, New Zealand
South America	Argentina, Brazil, Bolivia, Colombia, Peru, Venezuela

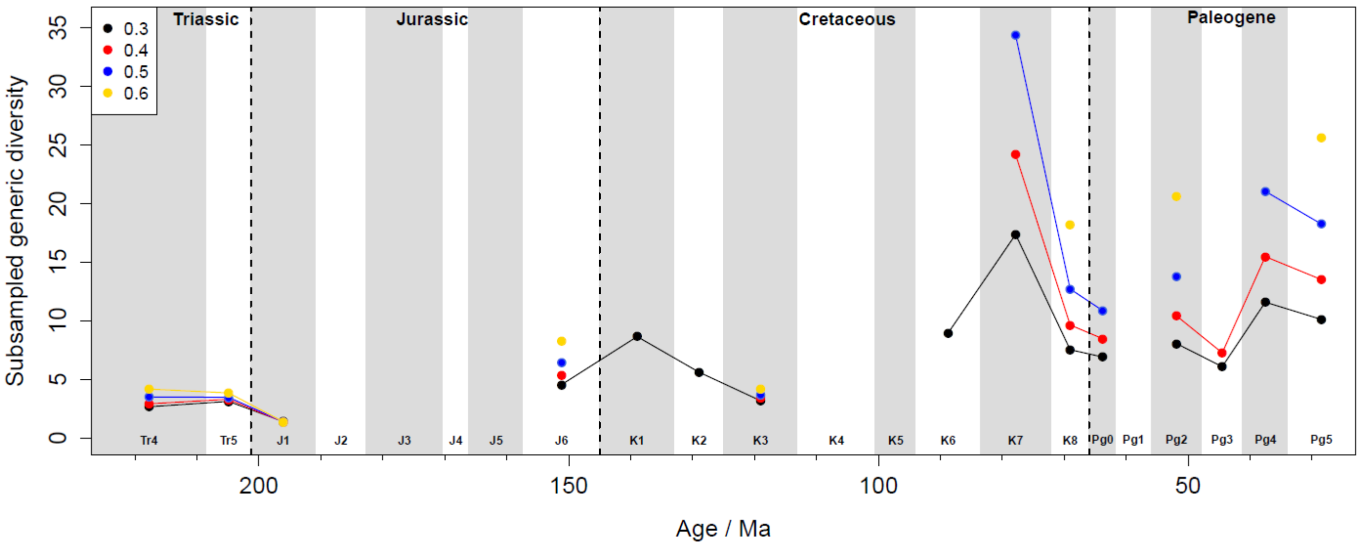

Figure S1. Subsampled global lepidosaur generic diversity from Triassic—Paleogene at quorum levels 0.4—0.6. Time bin durations are explained in Table 1.

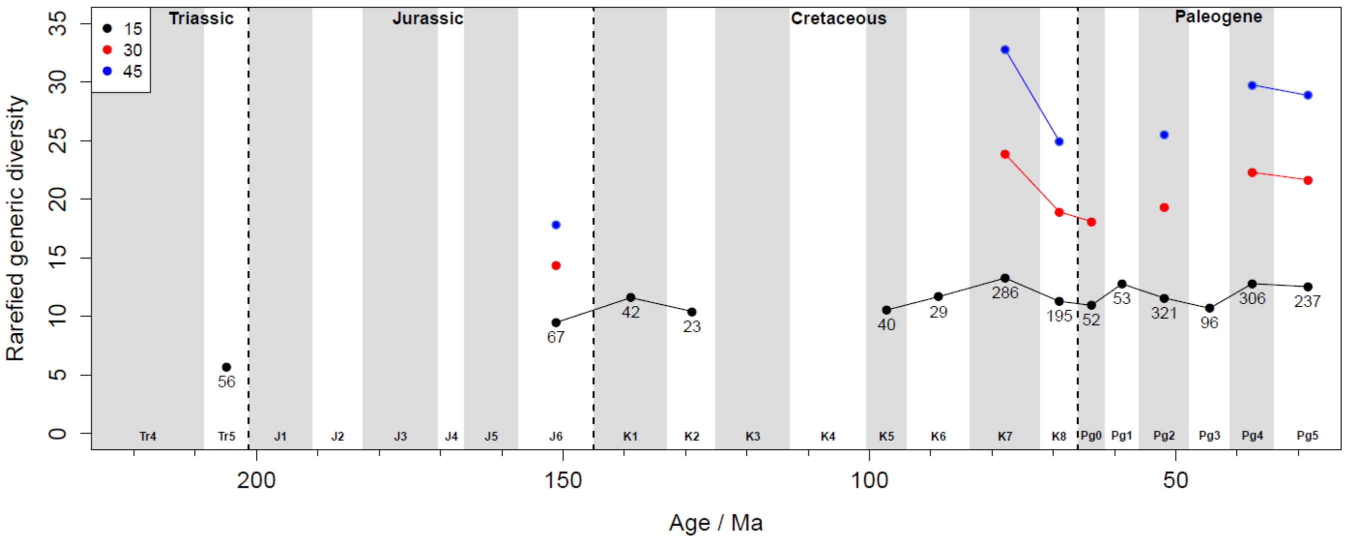

Figure S2. Rarefied global lepidosaur generic diversity from Triassic—Paleogene at a quota of 15, 30 and 45. Numbers indicate number of collections drawn for each time bin subsampled, as an indicator of underlying data quality. T, Triassic; J, Jurassic; K, Cretaceous; Pg, Paleogene.

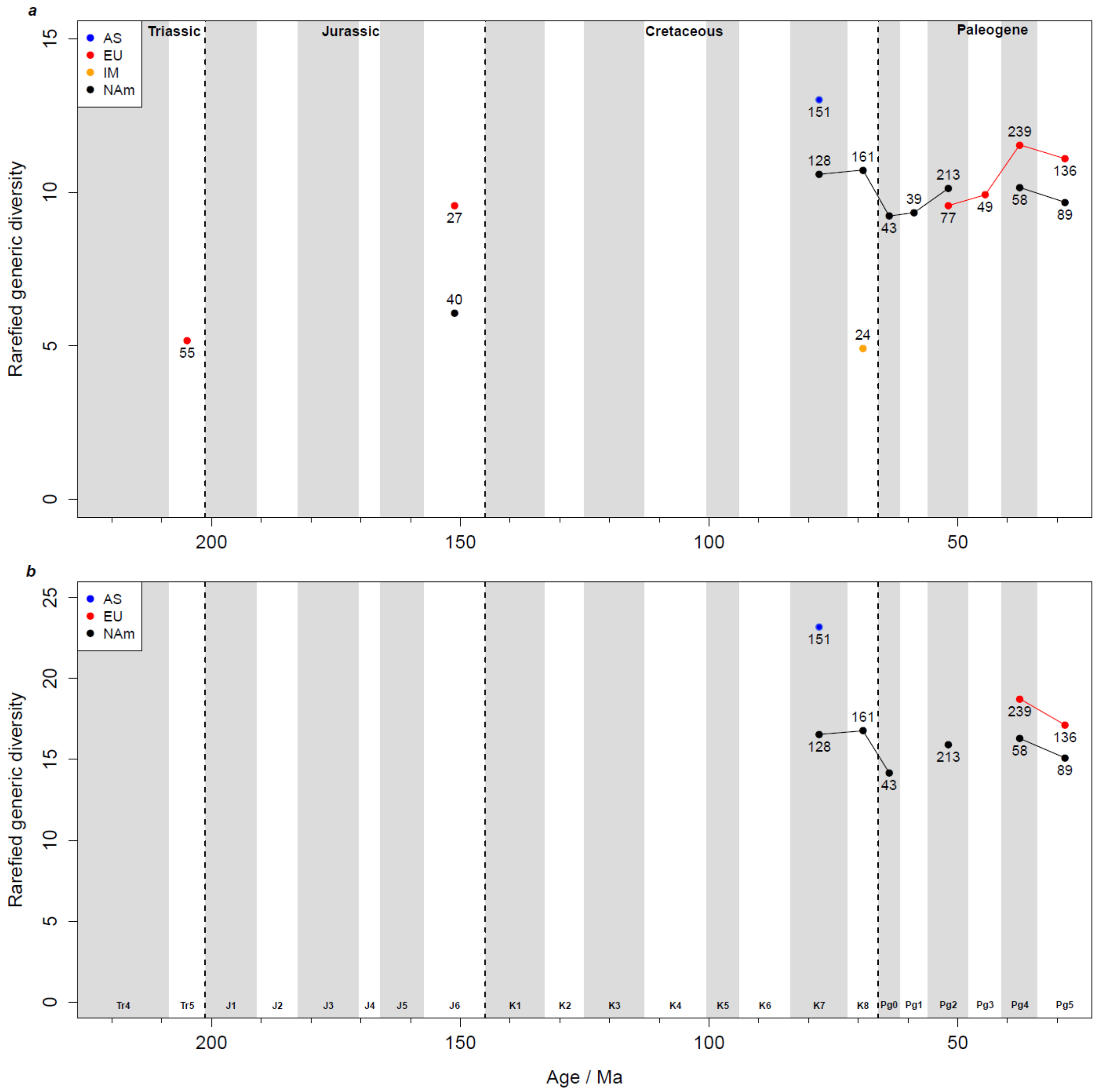

Figure S3. Rarefied terrestrial lepidosaur global generic diversity from Triassic—Paleogene using a quota of *(a)* 15 and *(b)* 30 for individual continents. AS, Asia; EU, Europe; IM, Indo-Madagascar, NAm, North America. Numbers indicate number of collections drawn for each time bin subsampled, as an indicator of underlying data quality. Time bin durations are explained in Table 1.

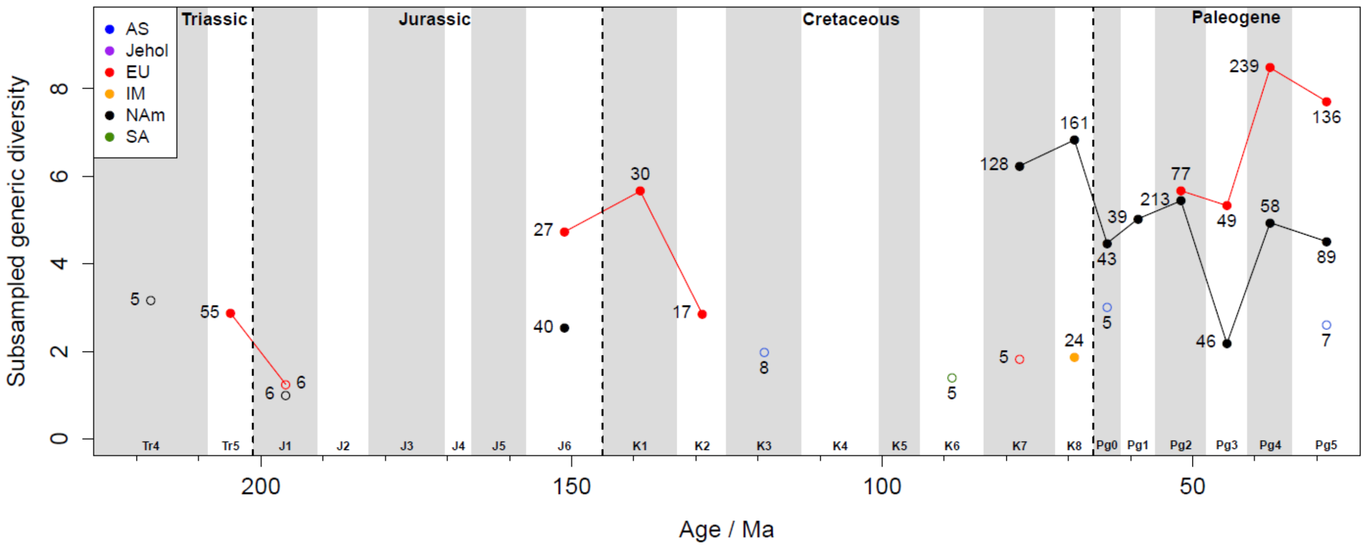

Figure S4. Subsampled terrestrial lepidosaur generic diversity from Triassic—Paleogene at quorum 0.3, for individual continents: AS, Asia; EU, Europe; IM, Indo-Madagascar, NAm, North America; SA, South America. Also included is the combined Jehol Group. Numbers indicate number of collections drawn for each time bin subsampled, as an indicator of underlying data quality. Time bin durations are explained in Table 1.

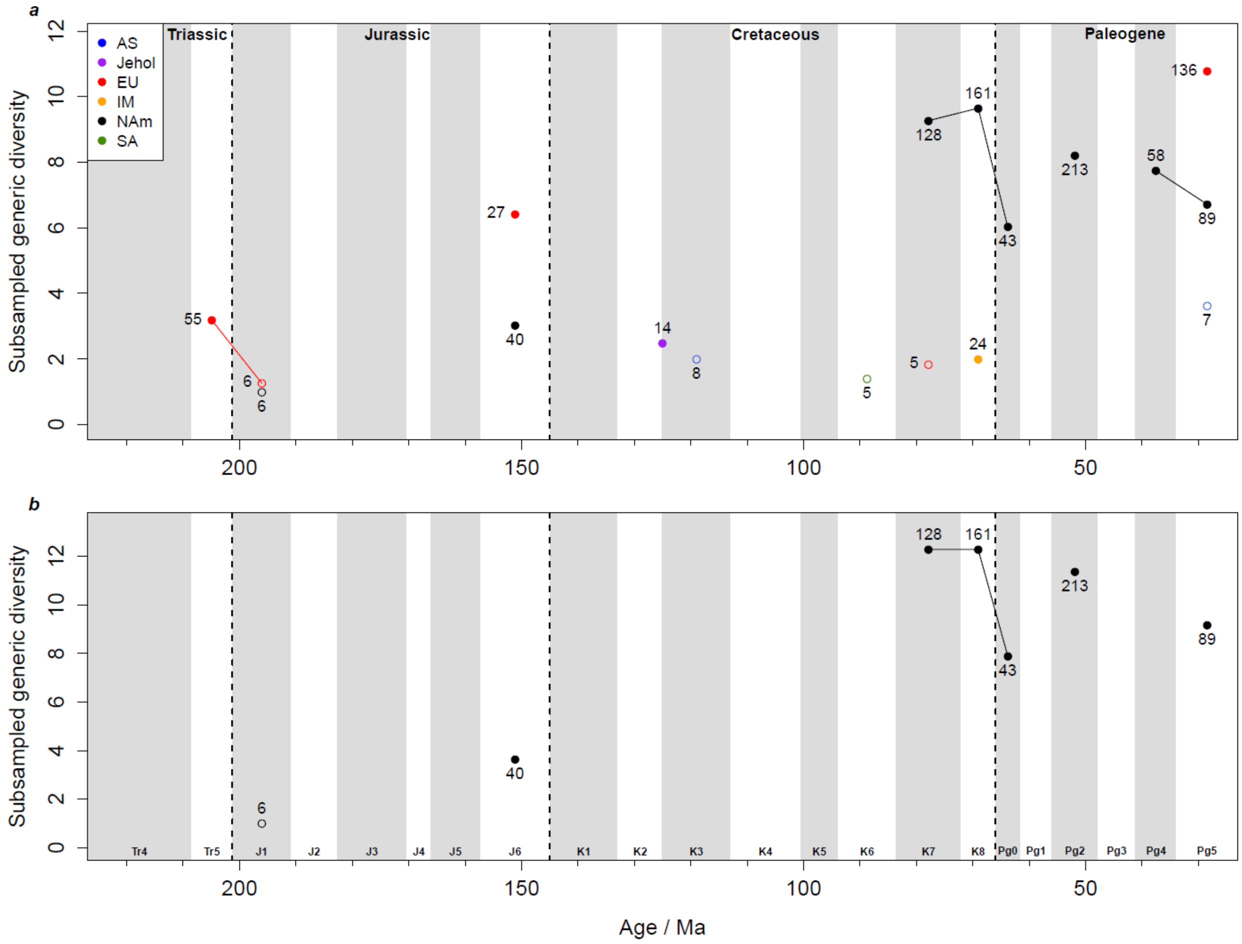

Figure S5. Subsampled terrestrial lepidosaur generic diversity from Triassic—Paleogene at quorum (a) 0.5, and (b) 0.6 for individual continents: AS, Asia; EU, Europe; IM, Indo-Madagascar, NAm, North America; SA, South America. Also included is the combined Jehol Group. Numbers indicate number of collections drawn for each time bin subsampled, as an indicator of underlying data quality. Time bin durations are explained in Table 1.

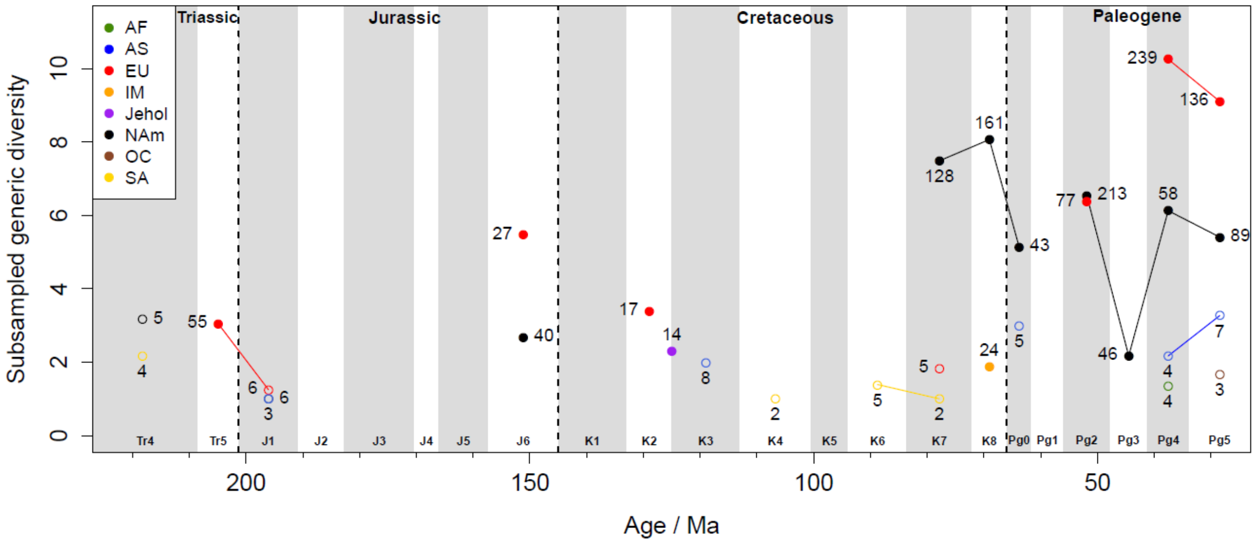

Figure S6. Subsampled terrestrial lepidosaur generic diversity from Triassic—Paleogene at quorum 0.4, for individual continents, including those points with fewer than 5 collections: AF, Africa; AS, Asia; EU, Europe; IM, Indo-Madagascar, NAm, North America; OC, Oceania; SA, South America. Also included is the combined Jehol Group. Numbers indicate number of collections drawn for each time bin subsampled, as an indicator of underlying data quality. Time bin durations are explained in Table 1.

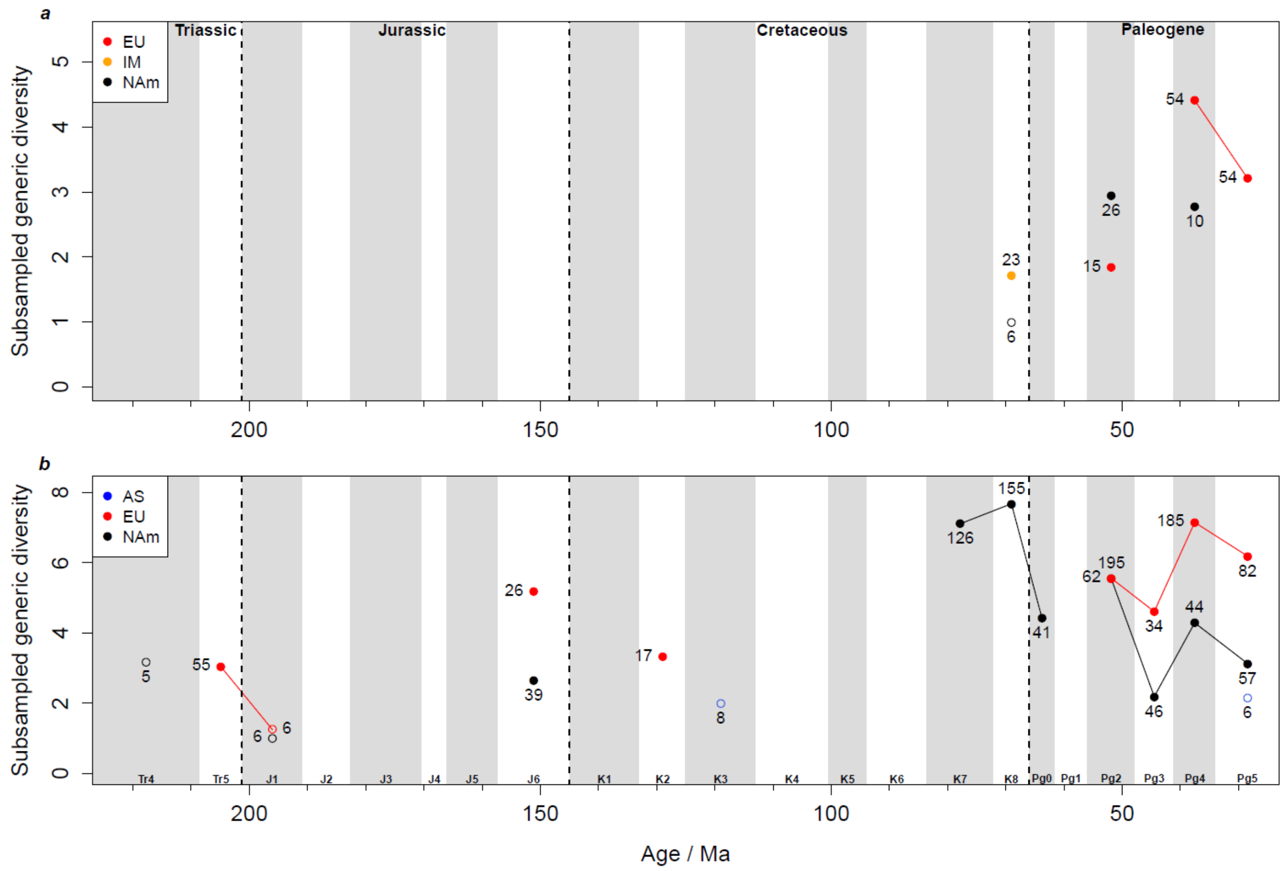

Figure S7. Subsampled generic diversity at quorum 0.4 for (a) terrestrial snakes and (b) terrestrial lepidosaurs excluding snakes for individual continents: AS, Asia; EU, Europe; IM, Indo-Madagascar, NAm, North America. Numbers indicate number of collections drawn for each time bin subsampled, as an indicator of underlying data quality. T, Triassic; J, Jurassic; K, Cretaceous; Pg, Paleogene. Time bin durations are explained in Table 1.
